# Statins and the risk of type 2 diabetes mellitus: cohort study using the UK clinical practice pesearch datalink

**DOI:** 10.1186/1471-2261-14-85

**Published:** 2014-07-15

**Authors:** Ana Filipa Macedo, Ian Douglas, Liam Smeeth, Harriet Forbes, Shah Ebrahim

**Affiliations:** 1 Department of Non-communicable Disease Epidemiology, London School of Hygiene and Tropical Medicine, Keppel Street, London WC1E 7HT, UK; 2 Faculty of Health Sciences, University of Beira Interior, Covilhã, Portugal

**Keywords:** Statins, Type 2 diabetes, Cardiovascular, Safety, Observational

## Abstract

**Background:**

There is strong evidence of reductions in major vascular events from statins across all cardiovascular risk categories. However, trials of statin therapy have provided conflicting results regarding statin use and type 2 diabetes (T2DM). We aimed to assess the effect of statins on T2DM development.

**Method:**

We carried out a population-based cohort study using the Clinical Practice Research Datalink (CPRD), a database of computerized clinical records. Every patient aged 30–85 years old starting a statin between 1989 and 2009 was matched with up to five non-statin users. The observation period in CPRD ended in 31 December 2011. Cox proportional hazard regression was used to compare rates of T2DM between statin users and non-users, using propensity score method to adjust for systematic differences between groups.

**Results:**

The study basis comprised 2,016,094 individuals, including 430,890 people who received a statin, matched to 1,585,204 people not prescribed a statin. Mean follow-up time was 5.43 years for statin users and 3.89 years for nonusers. During follow-up 130,395 individuals developed T2DM. Statin use was associated with an increased risk of T2DM (HR 1.57; 95% CI 1.54-1.59), which increases with longer duration of use. The increased risk was smaller among people with hypertension or cardiovascular disease and was only apparent after 5 or more years treatment with statins in these groups. Conversely, age-specific risk ratios decreased in older people.

**Conclusions:**

Statin use is associated with an increased risk of T2DM. Our results suggest that the relative risk is higher among people without diagnosed hypertension or cardiovascular disease. These findings should be considered in the context of the observational nature of the data which is prone to bias and unmeasured confounding.

## Background

Cardiovascular diseases (CVD) are the leading cause of premature death and a major cause of morbidity worldwide [1]. Reducing high blood cholesterol, a risk factor for CVD events, is recommended as part of the global risk management strategy for CVD prevention, with statins being widely used as first-choice lipid-lowering therapy after health behaviour interventions [2]. There is strong evidence of reductions in major vascular events from statins across all risk categories, from secondary prevention to those at moderate and low risk of vascular events in primary prevention [3-6].

Recently, two meta-analyses have raised concerns regarding the potential risk for developing type 2 diabetes (T2DM) during statin use (odds ratio 1.09; 95% CI 1.02-1.17 and odds ratio 1.09; 95% CI 1.02-1.16, respectively) [7,8]. The first study (West of Scotland Coronary Prevention Study [WOSCOPS]) that evaluated this outcome reported a protective effect (hazard ratio 0.70; 95% CI 0.50-0.98) but used no standardized criteria for T2DM diagnosis [9]. The increase in T2DM relative risk of 25% over a mean follow up of 1.9 years (hazard ratio 1.25; 95% CI 1.05-1.54) among participants in the JUPITER trial of the role of rosuvastatin in primary prevention reignited concern about this association [10]. Other studies have provided conflicting results regarding statin use and T2DM, including a nested case–control study using data from the UK General Practice Research Database (GPRD) that reported no strong evidence of a harmful effect of statins on the development of T2DM [11-13]. New analyses using data from the Women's Health Initiative (WHI) study suggest that the risk of T2DM among elderly women who reported statin use at baseline and at year 3 follow-up is higher than that observed in previous studies (HR = 1.47; 95% CI 1.32-1.64) [14]. In the Taiwan National Health Insurance Research Database, statin use increased the hazards of diabetes occurrence by 15% (HR 1.15; 95% CI 1.08 - 1.22) [15]. Furthermore, it appears that the risk of statin-induced diabetes is higher with intensive-dose statin therapy and among elderly people [6,16]. In Ontario Drug Benefit database patients treated with atorvastatin were found to have a 22% increased risk of new-onset diabetes, rosuvastatin an 18% increased risk and simvastatin a 10% increased risk, relative to pravastatin (reference group) [17]. The risk of developing diabetes on statins is strongly associated with baseline fasting blood glucose and with the number of co-existing CVD risk factors, suggesting that statins raise blood glucose by a small amount, moving people from below to above the diagnostic threshold [18]. However, a definitive mechanism by which statins increase incident diabetes has not yet been elucidated.

This issue raises new concerns on the benefit-risk ratio of statins in low cardiovascular risk individuals. The underlying incidence rates for T2DM vary between populations and have been rising rapidly worldwide over the past three decades [19,20]. Since 1990 the rising prevalence of type 2 diabetes, combined with a constant relative risk for CVD, has translated into a 60% increase in the attributable risk of CVD associated with diabetes, even while the attributable risk for CVD associated with other risk factors (hypertension and smoking) has held constant or fallen [21].

Quantifying the absolute effects of statins on T2DM is required to guide their clinical use. It is also necessary to determine how the results from clinical trials compare with what occurs in clinical practice, particularly among patients who are older, have more comorbid conditions, or receive higher statin doses than most patients in clinical trials. Therefore, use of large-scale observational data from routine medical practice to study the association between statin use and risk of T2DM is helpful in testing the hypothesis further. We therefore aimed to undertake a population-based cohort study based on computerized medical records derived from primary care in the UK to compare the rates of incident T2DM between users and non-users of statins.

## Method

We carried out a population-based retrospective cohort study using the Clinical Practice Research Datalink (CPRD).

### Ethics statement

This study was approved by the London School of Hygiene and Tropical Medicine Ethics Committee (application ref. 6081).

### Clinical practice research datalink

The Clinical Practice Research Datalink is a collection of anonymised longitudinal electronic health records from primary care in the United Kingdom (UK) hosted by the UK Medicines and Healthcare products Regulatory Agency’s (MHRA). All the data from patients is anonymised and no personal details are shared with health researchers. Data collection started in 1987. It includes diagnostic and prescribing information, as well as information on various lifestyle characteristics for more than 11 million patients from more than 600 general practices. The database is broadly representative of the UK population [22] and several studies have confirmed the validity of the diagnostic and prescription data in the CPRD for pharmacoepidemiological research [23-27].

### Participants

The source population was all patients registered with a general practice between January 1989 (statins were available from around this time and the database was started in 1987) and December 2009. Within this time period, study start dates were derived from the latest of the date the practice began contributing research quality data (CPRD defined quality marker based on assessment of completeness, continuity and plausibility of data recording in key areas) or the patient’s first registration date. Study end dates were derived using the earlier of the patient’s transfer out date or the practice’s last collection date.

We included in the study all patients aged 30–85 years old who received their first prescription for a statin on or after 1st January 1989 up until 31st December 2009, after at least 12 months registration in the CPRD to avoid including people who had been initiated on statin therapy prior to inclusion in the CPRD. For this study, the observation period in CPRD ended in 31 December 2011. Statin use was defined as at least one prescription during the study period and the date of first statin prescription was termed the index date. Then, we matched up to five non-statin users to each statin user on sex, general practice, age (+/- 2.5 years) and being registered in the CPRD on the index date of the statin user and for at least 12 months previously. Matching on general practice and index date helps reduce confounding by time period and by practice specific factors that might be difficult to measure or control for.

The index date for non-users was set as the index date for their matched statin user. Non-users also had to have no record of a statin prescription before the index date, but could be prescribed a statin later, in order to avoid selecting a biased control group who were not at risk of being prescribed a statin. We required both users and non-users of statins to have had some form of contact recorded with the practice within 12 months before and after the index date, in order to ensure all participants were active within their general practices. To avoid immortal time bias, we also took into account the time during which the outcome couldn’t occur for statin users. So, we included the period of time between cohort entry (study start dates) and the day before starting a statin in the pool of non-users that were matched with the users.

Patients were excluded from the study if they had a diagnosis of T2DM or T1DM at or before the index date. Pregnant women (10 months before the index date) or with gestational DM history were also excluded.

Incident T2DM diagnoses were ascertained from the CPRD record using Read codes (Additional file 1: list S1) and the date of diagnosis was determined by the earliest date of any recorded T2DM code. We censored follow-up at the earliest date of the diagnosis of T2DM, death from any cause, end of observation period in the CPRD or the date the study ended (31 December 2011).

### Analysis

#### Propensity score

In clinical practice exposure to statins is not randomized but is determined by a wide range of health-related factors. Systematic differences between statin users and non-users can bias the estimated exposure effect. Propensity scores (PS) can be used to adjust for the conditional probability of being prescribed a statin, given a set of chosen covariates. PS were estimated using conditional logistic regression, with statin prescription at the index date as the outcome. Variables were selected for inclusion in the PS model if they were associated with both the exposure and the outcome, or associated with the outcome only [28]: hyperlipidaemia, hypertension, abnormal glucose levels, smoking and drinking habits, family history of diabetes, family history of CVD, consultation rate (defined as the number of times a patient initiated contact with a general practice in the 12 months prior to the index date), prescribing rate (defined as the number of prescriptions in the 12 months prior to the index date), cardiovascular disease (including coronary heart disease, cerebrovascular disease and peripheral vascular disease), other unspecified atheroma, atrial fibrillation, heart failure, cancer, osteoporosis, recent hepatic or renal disease (within 6 months), thyroid disease, recent use of non-statin lipid-lowering medication (within 3 months), hormone replacement therapy (within 1 year), antidepressants (within 6 months), atypical antipsychotics (within 6 months), oral or inhaled glucocorticoids (within 6 months), bisphosphonates (within 1 year), cytochrome P450 3A4 inhibitors (within 3 months); and any previous use of cardiovascular drugs (including β-blockers, diuretics, calcium antagonists, angiotensin converting enzyme inhibitors, angiotensin receptor blockers, aspirin, anticoagulants, digoxin and nitrates).

We examined the distributions of PS in both exposure groups to ensure that only patients with overlapping scores were included. Patients with scores in the extreme upper and lower tail of the PS distribution (outside the 5th to the 95th percentiles) were excluded, as the inclusion of people treated contrary to extreme scores can introduce bias from unmeasured confounding [29]. Sensitivity analyses were conducted without this exclusion.

### Statistical analysis

All data management and statistical analysis were performed using STATA software.

In the primary analysis, Cox proportional hazard regression was used to compare rates of T2DM between statin users and non-users. Hazard ratios (HR) and 95% confidence intervals were estimated for statins initiated at index date, analogous to an intention to treat analysis. The initial model was adjusted for age, sex, family history of diabetes, post-index date new diagnosis of hepatic disease and propensity score categories. Further adjustment for sex, family history of diabetes and post-index date new diagnosis of hepatic disease did not affect the results, so these factors were not included. The final model included propensity score and further adjustment for age only.

Several sensitivity analyses were then carried out. First, we assessed the effect of censoring observation periods for each individual at the time they stopped taking statins and at the time their exposure status changed: when subjects changed their statin dose and/or switched to another statin and/or received another lipid-lowering drug in addition to the statin; or when subjects in the unexposed group were prescribed a statin. The date of stopping statins was assumed as 90 days after the date of the last recorded prescription. Secondly, we restricted the analysis to the first 6 months of exposure, since a positive association over a short exposure duration could indicate a possible bias. Missing data were quantified for each variable and classified as “unknown” category. For the majority of variables data was considered to be missing at random, so the main analyses didn’t exclude patients with missing data for any of the confounding variables. When the nature or the extent of missingness was considered important (e.g. BMI) we conducted a sensitivity analysis restricted to patients with low or normal BMI levels to explore its impact on the results.

As secondary analyses, to assess the possible effect modification of variables related to both the exposure and the outcome, we repeated the analyses stratified on age groups, body mass index (BMI) categories, baseline diagnoses of hypertension and CVD (including coronary heart disease, cerebrovascular disease and peripheral vascular disease), and duration of use (1 year, 1–3 years, 3–5 years, 5–10 years, 10–15 years, 15–20 years and 20–25 years).

## Results

The study basis comprised 2,016,094 individuals, including 430,890 people who received a statin, matched to 1,585,204 people not prescribed a statin (an average of 3.8 non-users per user). Mean follow-up time was 5.43 years (SD 3.08; maximum 21.76 years) for statin users and 3.89 years (SD 2.56; maximum 21.75 years) for nonusers.

Table 1 shows the baseline characteristics of statin users and non-users. Men and women were approximately equally represented. Compared with non-users of statins, new users tended to be older, were more overweight (67.51% with BMI > 25Kg/m^2^) and were more likely to have comorbidities, mainly hypertension and cardiovascular disease. They were also more likely to have had some form of contact with their practices and some drug prescription in the 12 months prior to the index date, as shown by their higher annual consultation and prescription rates. There were some missing data on smoking status (4.0%), alcohol consumption (11.7%), and BMI (12.5%).

**Table 1:** Baseline characteristics of study population of new users and non-users of statins

	**Exposed (new users)**	**Unexposed**
**Baseline characteristics**	**n (%)**	**n (%)**
**All patients (n = 2,016,094)**	**430,890 (21.37)**	**1,585,204 (78.63)**
**Gender**		
Male	231,732 (53.78)	835,053 (52.68)
Female	199,158 (46.22)	750,151 (47.32)
**Age mean (sd), yrs**	63.77 (10.81)	61.86 (11.27)
30-39	7,584 (1.76)	37,221 (2.35)
40-49	37,386 (8.68)	183,876 (11.60)
50-59	100,079 (23.23)	455,649 (28.74)
60-69	146,431 (33.98)	490,132 (30.92)
70-79	109,894 (25.50)	311,974 (19.68)
80-85	29,516 (6.85)	106,352 (6.71)
**BMI, kg/m**^**2**^		
<20	11,223 (2.60)	65,496 (4.13)
20-25	107,711 (25.00)	457,240 (28.84)
>25	290,876 (67.51)	831,395 (52.45)
Unknown	21,080 (4.89)	231,073 (14.58)
**Smoking status**		
Current smokers	90,670 (21.04)	266,333 (16.80)
Former smoker	163,576 (37.96)	532,087 (33.57)
Non-smoker/never	175,360 (40.70)	706,908 (44.59)
Unknown status	1,284 (0.30)	79,876 (5.04)
**Alcohol use**		
Current (unknown amount)	21,009 (4.88)	43,762 (2.76)
Rare drinker (<2u/d)	63,213 (14.67)	229,758 (14.49)
Moderate drinker (3-6u/d)	223,242 (51.81)	757,940 (47.81)
Excessive drinker (>6u/d)	40,912 (9.49)	135,790 (8.57)
Past use	8,130 (1.89)	40,337 (2.54)
Non-drinker	53,566 (12.43)	161,639 (10.20)
Unknown status	20,818 (4.83)	215,978 (13.62)
**Family history of:**		
Diabetes	17,914 (4.16)	55,962 (3.53)
Cardiovascular disease	113,104 (26.25)	307,406 (19.39)
**Diagnosis of:**		
Hyperlipidaemia	29,140 (6.76)	30,277 (1.91)
Atherosclerosis	1,664 (0.39)	2,175 (0.14)
Hypertension	176,946 (41.07)	352,371 (22.23)
Heart failure	10,448 (2.42)	20,160 (1.27)
Cardiovascular disease	98,739 (22.92)	101,666 (6.41)
Atrial fibrillation	17,907 (4.16)	36,005 (2.27)
Hepatic disease (within 6 months)	1,808 (0.42)	5,736 (0.36)
Kidney disease (within 6 months)	13,813 (3.21)	27,352 (1.73)
Abnormal glucose level	6,076 (1.41)	11,757 (0.74)
Osteoporosis	13,047 (3.03)	45,262 (2.86)
Cancer	42,929 (9.96)	159,664 (10.07)
Thyroid disease	34,863 (8.09)	105,555 (6.66)
**Prior use of:**		
Antipsychotics (within 6 months)	252 ( 0.06)	1,317 (0.08)
Antidepressants (within 6 months)	5,876 (1.36)	15,495 (0.98)
Cardiovascular drugs	124,687 (28.94)	608,423 (38.38)
Nonstatin lipid lowerers (within 3 months)	894 (0.21)	336 (0.02)
Systemic glucocorticoids (within 6 months)	5,617 (1.30)	14,354 (0.91)
Oestrogens/HRT (within 1 year)	3,222 (0.75)	6,265 (0.40)
Bisphosphonates (within 1 year)	2,310 (0.54)	10,154 (0.64)
CYP450 3A4 inhibitor	6,962 (1.62)	13,363 (0.84)
**Annual consultation rate, mean (SD)**	25.99 (18.56)	15.76 (17.42)
**Annual prescription rate, mean (SD)**	25.80 (31.69)	8.34 (21.02)

All the differences between groups were significant at the 0.05 level (two-sided).

Of the new statin users, 271,126 (62.92%) had been prescribed simvastatin; 111,734 (25.93%) atorvastatin; 25,881 (6.00%) pravastatin; 15,380 (3.57%) rosuvastatin and 5,963 (1.38%) fluvastatin (Table 2). Additionally, 720 (0.17%) individuals were prescribed simvastatin with ezetibime and 86 (0.02%) received a statin and another lipid lowering drug.

**Table 2:** Baseline characteristics of patients who developed T2DM

**Baseline characteristics**	**Subjects with incident T2DM**	**Subjects without incident T2DM**
	**n (%)**	**n (%)**
**All patients (n = 2,016,094)**	**130,395 (6.47)**	**1,885,699 (93.53)**
**Gender**		
Male	70,972 (54.43)	995,813 (52.81)
Female	59,423 (45.57)	889,886 (47.19)
**Age mean (sd), yrs**	63.44 (10.54)	62.19 (11.24)
30-39	1,697 (1.30)	43,108 (2.29)
40-49	11,324 (8.68)	209,938 (11.13)
50-59	33,703 (25.85)	522,025 (27.68)
60-69	43,636 (33.46)	592,927 (31.44)
70-79	32,174 (24.67)	389,694 (20.67)
80-85	7,861 (6.03)	128,007 (6.79)
**BMI, kg/m**^**2**^		
<20	2,461 (1.89)	74,258 (3.94)
20-25	20,947 (16.06)	544,004 (28.85)
>25	102,001 (78.22)	1,020,270 (54.11)
Unknown	4,986 (3.82)	247,167 (13.11)
**Smoking status**		
Current smokers	21,742 (16.67)	335,261 (17.78)
Former smoker	59,403 (45.56)	636,260 (33.74)
Non-smoker/never	48,775 (37.41)	833,493 (44.20)
Unknown status	475 (0.36)	80,685 (4.28)
**Alcohol use**		
Current (unknown amount)	5,044 (3.87)	59,727 (3.17)
Rare drinker (<2u/d)	21,939 (16.83)	271,032 (14.37)
Moderate drinker (3-6u/d)	64,639 (49.57)	916,543 (48.60)
Excessive drinker (>6u/d)	40,912 (9.49)	135,790 (8.57)
Past use	4,200 (3.22)	44,267 (2.35)
Non-drinker	17,844 (13.68)	197,361 (10.47)
Unknown status	5,809 (4.45)	230,987 (12.25)
**Family history of:**		
Diabetes	6,607 (5.07)	67,269 (3.57)
Cardiovascular disease	29,012 (22.25)	391,498 (20.76)
**Diagnosis of:**		
Hyperlipidaemia	6,372 (4.89)	53,045 (2.81)
Atherosclerosis	373 (0.29)	3.466 (0.18)
Hypertension	50,529 (38.75)	478,788 (25.39)
Heart failure	3,235 (2.48)	27,373 (1.45)
Cardiovascular disease	20,852 (15.99)	179,553 (9.52)
Atrial fibrillation	5,117 (3.92)	48,795 (2.59)
Hepatic disease (within 6 months)	787 (0.60)	6,757 (0.36)
Kidney disease (within 6 months)	2,671 (2.05)	38,494 (2.04)
Abnormal glucose level	3,780 (2.90)	14,053 (0.75)
Osteoporosis	3,099 (2.38)	55,210 (2.93)
Cancer	12,916 (9.91)	189,677 (10.06)
Thyroid disease	10,747 (8.24)	129,671 (6.88)
**Prior use of:**		
Antipsychotics (within 6 months)	114 ( 0.09)	1,455 (0.08)
Antidepressants (within 6 months)	1,503 (1.15)	19,868 (1.05)
Cardiovascular drugs	53,096 (40.72)	680,014 (36.06)
Nonstatin lipid lowerers (within 3 months)	189 (0.14)	1,041 (0.06)
Systemic glucocorticoids (within 6 months)	1,496 (1.15)	18,475 (0.98)
Oestrogens/HRT (within 1 year)	706 (0.54)	8,781 (0.47)
Bisphosphonates	699 (0.54)	11,765 (0.62)
CYP450 3A4 inhibitor	1,709 (1.31)	18,616 (0.99)
**Annual consultation rate, mean (SD)**	22.16 (19.33)	17.66 (18.04)
**Annual prescription rate, mean (SD)**	19.42 (30.12)	11.57 (24.27)

All the differences between groups were significant at the 0.05 level (two-sided).

During follow-up 130,395 individuals developed T2DM, 56,702 cases among statin users and 73,693 among non users. Patients who developed T2DM were older, were more overweight (78.22% with BMI > 25Kg/m^2^) and were more likely to have comorbidities, mainly hypertension and cardiovascular disease (Table 2). The severity of T2DM, measured by the type of treatment used, differed by statin exposure status: six months after T2DM diagnosis 41,210 patients (21.40% users vs. 39.95% non users) were being prescribed oral anti-diabetic drugs (OAD) and 4,439 patients (0.25% users and 4.55% non users) were being prescribed a combination of OAD and insulin. The remaining 84,746 patients (77.34% users and 55.49% non users) with T2DM were not being prescribed any hypoglycemic medicine 6 months after diagnosis.

Table 3 shows the crude incidence of T2DM per 1000 person-years by statin exposure status, crude and adjusted hazard ratios. Hazard ratios were increased in crude, adjusted and fully adjusted models with little attenuation; after adjustment for age and propensity score (final model) statin use was associated with an increased risk of T2DM (HR 1.57; 95% CI 1.54-1.59). In this primary analysis restricted to the 5th-95th percentile of PS distribution the cohort consisted of 1,448,993 individuals, of whom 386,746 were users and 1,062,247 nonusers. The sensitivity analysis conducted without this restriction gave similar results. The hazard ratios were higher in the first 6 months of exposure.

**Table 3:** Incidence rates of T2DM by statin exposure status and crude and adjusted hazard ratios

	**Nº of subjects**	**Incident T2DM per 1000 person-years (95% CI)**		**Hazard ratio (95% CI) crude**	Hazard ratio (95% CI) fully adjusted*	Hazard ratio (95% CI) adjusted for age and propensity score**
		**Exposed**	**Unexposed**			
**All sample**	2,016,094	24.24 (24.04-24.44)	11.95 (11.86-12.04)	1.99 (1.97-2.01)	1.57 (1.54-1.59)	1.57 (1.55-1.60)
**PS [5-95%]**	1,448,993	23.90 (23.69-24.11)	12.2 (12.01-12.31)	1.91 (1.89-1.94)	1.56 (1.54-1.59)	1.57 (1.54-1.59)
**Stop**	2,016,094	25.32 (25.71-26.14)	11.95 (11.86-12.04)	2.13 (2.11-2.16)	1.70 (1.67-1.72)	1.71 (1.68-1.73)
	1,448,993	25.54 (25.32-25.77)	12.20 (12.10-12.31)	2.05 (2.02-2.07)	1.69 (1.66-1.71)	1.70 (1.67-1.72)
**Change**	2,016,094	29.38 (29.14-29.63)	12.13 (12.04-12.21)	2.42 (2.40-2.45)	1.90 (1.87-1.93)	1.91 (1.88-1.93)
	1,448,993	28.98 (28.73-29.24)	12.39 (12.28-12.50)	2.33 (2.31-2.36)	1.90 (1.87-1.93)	1.91 (1.88-1.94)
**First**	2,016,094	23.80 (23.15-24.47)	9.69 (9.48-9.91)	2.45 (2.37-2.54)	2.15 (2.05-2.25)	2.14 (2.05-2.25)
**6 months**	1,448,993	23.92 (23.23-24.62)	9.86 (9.59-10.13)	2.43 (2.33-2.52)	2.18 (2.08-2.29)	2.18 (2.07-2.29)
**After**	2,003,499	24.28 (24.07-24.89)	12.28 (12.37-24.49)	1.95 (1.92-1.97)	1.51 (1.49-1.54)	1.52 (1.49-1.54)
**6 month**	1,436,377	23.89 (23.68-24.11)	12.54 (12.43-12.66)	1.86 (1.84-1.89)	1.50 (1.47-1.53)	1.51 (1.48-1.53)

PS – propensity score; Stop – observation periods censored at time of statin withdrawn Change – observation periods censored at time exposure status changed.

All the variables in Table 1 were included in propensity score model. *Full model: adjusted for age, gender, propensity score, post index date diagnosis of hepatic disease and family history of diabetes. **Final model: adjusted for age and propensity score.

During the observation period, 14.46% (n = 62,301) of the exposed group stopped statin therapy and 8.43% (n = 169,900) of the participants changed their treatment. The re-analysis censoring observation periods at the time of stopping statin exposure or changing treatment found small increases in rates, particularly in the exposed group, with consequently higher hazard ratios.

To assess the possible effect modification of variables related to both the exposure and the outcome, we repeated the analyses stratified by age groups, BMI categories, hypertension and CVD diagnoses, and duration of statin use (Table 4). The stratified analysis found increased risks of T2DM associated with higher BMI levels and longer follow-up. Conversely, age-specific risk ratios decreased in older people, even though an association was observed in all strata. Incidence rates of T2DM increase with age in non-statin users, whereas in statin users incidence rates were higher and did not show an age gradient.

**Table 4:** Risk of T2DM by statin use, stratified by age groups, BMI categories, hypertension, CVD and follow-up time

**PS [5-95%]**	**Incident T2DM per 1000 person-years (95% CI)**		**Hazard ratio (95% CI) adjusted for age and propensity score**
**(N = 1,448,993)**	**Exposed**	**Unexposed**	
**Age**			
30-39	23.16 (21.70-24.72)	3.89 (3.59-4.21)	4.68 (4.12-5.31)
40-49	25.13 (24.43-25.84)	6.70 (6.80-7.20)	2.84 (2.70-2.99)
50-59	25.22 (24.79-25.65)	10.83 (10.65-11.00)	1.87 (1.82-1.93)
60-69	23.88 (23.52-24.23)	14.53 (14.31-14.75)	1.34 (1.30-1.37)
70-79	22.94 (22.52-23.36)	17.81 (17.49-18.14)	1.15 (1.11-1.19)
80-85	20.30 (19.48-21.15)	13.83 (13.32-14.36)	1.25 (1.17-1.34)
**BMI, kg/m**^**2**^			
<20	11.71 (10.79-12.70)	6.76 (6.38-7.15)	1.20 (1.06-1.36)
20-25	13.22 (12.92-13.53)	6.54 (6.41-6.67)	1.51 (1.45-1.57)
>25	28.99 (28.72-29.75)	17.18 (17.01-17.35)	1.40 (1.38-1.43)
**Hypertension**	26.41 (26.06-26.77)	21.80 (21.49-22.12)	1.04 (1.01-1.07)
**No hypertension**	22.32 (22.06-22.57)	9.69 (9.58-9.80)	1.92 (1.88-1.95)
**CVD**	23.42 (23.00-23.85)	21.91 (21.32-22.53)	0.86 (0.82-0.89)
**No CVD**	24.04 (23.80-24.28)	11.63 (11.53-11.74)	1.70 (1.67-1.72)
**Follow up time (years)**			
0-1 yr	19.97 (19.52-20.42)	11.53 (11.33-11.74)	1.52 (1.47-1.57)
1-3 yr	20.11 (19.77-20.44)	13.55 (13.37-13.74)	1.22 (1.19-1.25)
3-5 yr	24.56 (24.12-25.00)	11.61 (11.38-11.84)	1.73 (1.68-1.80)
5-10 yr	30.04 (29.55-30.53)	10.85 (10.59-11.12)	2.25 (2.17-2.34)
10-15 yr	34.93 (33.52-36.39)	10.20 (9.38-11.10)	3.05 (2.71-3.43)
15-20 yr	42.24 (36.90-48.36)	12.24 (9.08-16.51)	3.63 (2.44-5.38)
20-25 yr	13.10 (1.84-93.00)	n.e.	n.e.

n.e - no estimation.

There was only a small increased risk of T2DM associated with statin exposure among people with diagnosed hypertension at baseline (HR 1.04; 95% CI 1.01-1.07); and there was evidence of a protective effect in people with diagnosed CVD at baseline (HR 0.86; 95% CI 0.82-0.89). To explore further this effect modification we stratified the analysis for these groups according to different follow-up times and calculated the median time for T2DM diagnosis in each time band (Table 5). The time varying analysis confirmed that the highest risk observed in the first year of starting treatment could be due to a detection bias, since among statin users 50% of T2DM diagnoses were done 139 days (4.5 months) after starting treatment, compared to 210 days (7 months) for the nonusers. This bias doesn’t seem to continue throughout statin use since no significant differences were observed in the other time periods. An increased hazard of T2DM among statin users was apparent from the first year among people without hypertension or CVD but in people with these conditions an increased hazard of T2DM was apparent by 5 years of use. In a sensitivity analysis to explore the impact of exposure status changing during follow-up we re-ran the analysis censoring observation periods at the time of stopping statin exposure or changing treatment. This showed no differences in risk estimates (Additional file 1: Table S1). Since diabetes, hypertension and CVD share similar underlying risk factors, we also compared the proportions of some baseline predictors of new-onset T2DM for hypertensive and CVD patients, according to their exposure status (Additional file 1: Table S2). No significant differences were observed according to exposure status. In all groups the majority of individuals were overweight or obese and a large proportion of CVD patients were hypertensive (33.98% in exposed vs. 50.58% in unexposed), indicating an overlap among the CVD and hypertension groups.

**Table 5:** Incident rates and median time for T2DM diagnosis at different follow-up times, according to hypertension and CVD diagnosis

		**Statin use**			
		**Yes**		**No**	
**Follow up time (years) PS [5-95%] (N = 1,448,993)**	Hazard ratio (95% CI)*	**Incident T2DM per 1000 person-years (95% CI)**	**Nº T2DM /Median time (days)**	**Incident T2DM per 1000 person-years (95% CI)**	**Nº T2DM /Median time (days)**
**Total sample**					
**0-1 yr**	1.52 (1.47-1.57)	19.97 (19.52-20.42)	7,634/139	11.53 (11.33-11.74)	12,189/210
**1-3 yr**	1.22 (1.19-1.25)	20.11 (19.78-20.44)	13,786/727	13.35 (13.34-13.37)	21,418/644
**3-5 yr**	1.73 (1.68-1.80)	24.56 (24.12-25.00)	11,858/1,440	11.61 (11.38-11.84)	9,773/1,394
**5-10 yr**	2.25 (2.17-2.34)	30.03 (29.55-30.53)	14,523/2,450	10.85 (10.59-11.12)	6,534/2,312
**10-15 yr**	3.05 (2.71-3.43)	34.93 (33.53-36.39)	2,292/4,174	10.20 (9.38-11.10)	543/4,166
**15-20 yr**	3.63 (2.44-5.38)	42.24 (36.90-48.36)	210/5,877	12.24 (9.10-16.51)	43/5,773
**20-25 yr**	n.e.	13.10 (1.84-93.00)	1/ -	0	0/ -
**No hypertension**					
**0-1 yr**	2.05 (1.96-2.15)	19.53 (18.97-20.11)	4,486/134	8.65 (8.44-8.85)	6,893/215
**1-3 yr**	1.50 (1.45-1.55)	18.64 (18.23-19.06)	7,694/727	10.60 (10.42-10.77)	13,114/653
**3-5 yr**	2.00 (1.92-2.09)	22.15 (21.61-22.69)	6,463/1,439	9.56 (9.33-9.80)	6,550/1,400
**5-10 yr**	2.50 (2.39-2.61)	27.65 (27.06-28.24)	8,502/2,482	9.32 (9.06-9.59)	4,750/2,335
**10-15 yr**	3.14 (2.75-3.58)	32.86 (31.26-34.55)	1,541/4,180	9.31 (8.48-10.21)	446/4,186
**15-20 yr**	3.84 (2.52-5.86)	41.61 (35.64-48.59)	160/5,877	11.55 (8.41-15.88)	38/5,780
**20-25 yr**	n.e.	0	0/ -	0	0/ -
**Hypertension**					
**0-1 yr**	0.96 (0.91-1.01)	20.62 (19.91-21.35)	3,148/145	20.40 (19.86-20.96)	5,296/204
**1-3 yr**	0.84 (0.81-0.88)	22.33 (21.77-22.89)	6,092/726	24.20 (23.68-24.72)	8,304/625
**3-5 yr**	1.22 (1.15-1.29)	28.24 (27.50-29.00)	5,395/1,442	20.51 (19.82-21.23)	3,223/1,380
**5-10 yr**	1.56 (1.46-1.67)	34.22 (33.37-35.10)	6,021/2,405	19.25 (18.38-20.17)	1,784/2,264
**10-15 yr**	2.19 (1.68-2.84)	40.09 (37.32-43.06)	751/4,156	18.33 (15.02-22.37)	97/4,073
**15-20 yr**	2.32 (0.77-7.01)	44.38 (33.64-58.56)	50/5,876	22.38 (9.31-53.77)	5/ -
**20-25 yr**	n.e.	0	0/ -	0	0/ -
**No CVD**					
**0-1 yr**	1.74 (1.67-1.80)	20.72 (20.22-21.24)	6,260/136	10.74 (10.54-10.95)	10,474/213
**1-3 yr**	1.31 (1.28-1.35)	20.37 (19.99-20.75)	11,037/725	12.93 (12.75-13.11)	19,254/647
**3-5 yr**	1.85 (1.78-1.92)	24.94 (24.44-25.45)	9,325/1,438	11.18 (10.95-11.42)	9,022/1,393
**5-10 yr**	2.34 (2.24-2.43)	30.44 (29.86-31.02)	10,512/2,416	10.56 (10.30-10.83)	6,134/2,317
**10-15 yr**	2.96 (2.61-3.35)	33.58 (31.85-35.97)	1,379/4,151	10.08 (9.26-10.99)	523/4,166
**15-20 yr**	3.76 (2.49-5.67)	41.78 (35.54-49.11)	147/5,985	11.81 (8.70-16.04)	41/5,773
**20-25 yr**	n.e.	0	0/ -	0	0/ -
**CVD**					
**0-1 yr**	0.75 (0.69-0.82)	17.11 (16.23-18.04)	1,374/147	21.05 (20.08-22.07)	1,715/196
**1-3 yr**	0.75 (0.70-0.80)	19.12 (18.42-19.85)	2,749/733	23.77 (22.79-24.79)	2,164/613
**3-5 yr**	0.97 (0.88-1.06)	23.24 (22.35-24.17)	2,533/1,445	21.30 (19.83-22.88)	751/1,408
**5-10 yr**	1.34 (1.19-1.50)	29.05 (28.16-29.96)	4,011/2,540	18.73 (16.98-20.66)	400/2,223
**10-15 yr**	2.26 (1.42-3.57)	37.19 (34.85-39.68)	913/4,224	14.70 (9.49-22.79)	20/4,204
**15-20 yr**	1.45 (0.31-6.74)	43.36 (33.87-55.08)	63/5,762	48.47 (12.12-193.81)	2/ -
**20-25 yr**	n.e.	0	0/ -	0	0/ -

PS – propensity score; *Final model: adjusted for age and propensity score; n.e. – no estimation.

To examine whether the lower and protective hazard ratios observed in patients with hypertension and CVD were due to their high BMI levels we repeated the analysis for these groups, including only patients with BMI ≤ 25Kg/m^2^ (underweight/normal) (Additional file 1: Table S3). The observed rates of T2DM across the exposed groups were similar among both hypertensives and non-hypertensives and among the CVD and no-CVD groups with increased incidence of T2DM occurring from 3–5 years of exposure and consistently increased hazard ratios after 5 years of use. These findings suggest that in non-overweight people long-term statin use may also increase occurrence of T2DM.

## Discussion

This study examined and quantified the risk of diabetes associated with statin use in a large representative primary care population over more than 20 years. We found an increased risk of T2DM associated with statin use, which increases with longer duration of use and higher baseline BMI levels, and decreases with age. The size of the increased risk was smaller among people with diagnosed hypertension or cardiovascular disease and was only evident after 5 or more years treatment in these groups.

Our findings confirm those from randomized trials where an increased risk of diabetes was found in both primary and secondary prevention (OR 1.18; 95% CI 1.01-1.39 and OR = 1.09; 95% CI 1.02-1.17 respectively) [7,29]. The Cholesterol Treatment Trialist Collaborators did not include diabetes among the adverse effects studied. Recently, one prospective cohort study of large sample size (Women’s Health Initiative) found evidence of an increased risk of T2DM (HR = 1.47; 95% CI 1.32-1.64) for the groups of women who reported statin use at baseline and at year 3 follow-up [14] This observational study, in common with our study, found a much stronger association than those found in clinical trials.

Our results also suggest an apparent effect modification by hypertension and CVD in the risk of T2DM associated with statin use. The baseline risk of T2DM observed in these groups (around 20 per 1000 person-years) is similar to the rates found in other studies [30,31] Statins do not seem to have any additional effect on this baseline risk of T2DM in the first years of treatment. After 5 years, statin use was associated with an increased risk of T2DM.

Several studies have demonstrated that persons with hypertension are at significantly elevated risk of developing Type 2 diabetes [31-35]. These studies have shown a close association between insulin resistance and hypertension, although the question whether the insulin resistance leads to hypertension or vice versa is not yet clear [36,37]. The mechanism by which statins increase incident diabetes is not known. In a systematic review and meta-analysis by Baker and colleagues, statins as a class had no impact on insulin sensitivity, though pravastatin increased insulin sensitivity and simvastatin (lipophilic statin) worsened it [38]. Statins may also directly affect insulin secretion via the convergence of multiple mechanisms that compromise the integrity and function of β-cells [39-41]. Kruit and colleagues have recently established a role between cell cholesterol homeostasis and insulin secretion [41-43]. The inhibition of HMG-CoA reductase upregulates the expression of LDL receptor in a time-dependent manner. In the liver, this leads to increased cholesterol clearance in bile. However, in peripheral tissues like pancreatic β-cells that do not degrade cholesterol, enhanced LDL receptor expression could lead to increased cellular cholesterol and impair insulin secretion [41-43]. A number of genes involved in lipid metabolism have also been implicated in T2DM, however further research is needed to extrapolate the findings to humans [41,42].

Our observed differences in risk of T2DM in people with or without hypertension or CVD in the first 5 years of treatment is surprising. It is likely that people started on statins and also diagnosed with hypertension and CVD will also receive more life-style advice, in particular increased physical activity, and may be more likely to adhere to advice. There is evidence that such interventions reduce the risk of T2DM [44], but may not provide long-term protection [45], which may explain our findings. Over time, impaired insulin secretion induced by statins worsens, more beta cells become unable to secrete sufficient insulin to compensate for insulin resistance with hyperinsulinemia, increasing the risk of developing Type 2 diabetes with longer statin use. This mechanism could make hypertensive patients, already with insulin resistance, develop T2DM faster than non-hypertensives, which would explain the slightly higher rates of T2DM observed in hypertensive statins users per time band, compared with non-hypertensives users.

The hazard ratios observed in patients with hypertension and CVD were not due to their high BMI levels (as shown by our sensitivity analysis), although small decreased rates of T2DM were found when the analysis was restricted to patients with low BMI levels (underweight/normal). Obesity and overweight are strong predictors of T2DM, with profound impact on the disease progression, often preceding the development of many metabolic disorders characterized by insulin resistance, including hypertension and CVD [46].

The age-specific risk ratios of T2DM decreased in older people. In non statin-users the increase in T2DM with age is expected probably due to age-dependent loss of β-cells and increased obesity and reduced physical activity. In statin users the T2DM rates were higher and did not show an age gradient. There is a growing consensus that one mechanism by which statins increase incident diabetes is by pushing people over the diagnostic threshold of blood glucose earlier than people with similar risk factor profiles but not taking statins. A further mechanism is that younger people at high risk of developing diabetes are accurately targeted for statin treatment, which in conjunction with the former effect would attenuate the expected age gradient. The progression of pre-diabetes to T2DM has been extensively studied and both higher BMI, weight gain, blood pressure, younger age and triglycerides have been identified as predictors of diabetes development [47-49]. The increased risk of developing T2DM with longer statin use is difficult to separate from the effect of other predictors of worsening beta cell function/decreased insulin sensitivity over time. T2DM is a multifactorial disease, with complex interactions between various environmental, behavioral and genetic factors, making the contribution of each single factor difficult to assess.

### Limitations and strengths

A major strength of this study was the large cohort size and the potential for long term follow-up, giving sufficient power to analyze comparatively rare and long latency outcomes such as T2DM. Additionally, our study was based on routine clinical data representative of the general UK population, and therefore better reflects the nature and frequency of events experienced in general clinical practice.

Despite these positive aspects, observational studies have limitations, notably bias and unmeasured confounding. Confounding was reduced by adjustment for a wide range of potential confounding factors and by the use of propensity scores and matching. Propensity score analysis can successfully account for much confounding by indication, but residual confounding is possible due to risk factors that have not been considered in the analyses, or to potential changes in risk factors during follow up.

Prescription data were prospectively recorded prior to the outcomes, with no potential for recall bias. Misclassification of exposure is possible because low dose of statins may be purchased over the counter. However, it is likely that most prescriptions are issued in primary care, especially among people aged 60+ and those with comorbidities, who will have free prescriptions. A prescription does not necessarily mean that a patient has taken a drug and non-adherence to treatment is likely to affect any study. Although adherence with statins could not be ascertained, 85% and 83% of statins users had at least one statin prescription between 12 and 24 months of follow up (year 2) and between 24 and 36 months (year 3), respectively. We believe that repeat prescribing for statins is likely to represent regular and genuine drug use in most cases. Misclassification of the outcome is possible, since we did not have information on the diagnostic criteria, although validation studies undertaken using GPRD data have shown high levels of accuracy [23-27], and any such misclassification is likely to be non-differential with respect to exposure, expected to lead to a reduction in power only. Misclassification of BMI due to missing data is possible. Although we had no information on the reasons for missing BMI, our findings didn’t change when our analysis was restricted to patients with low or normal BMI levels.

The marked increased risk of T2DM observed in the first year could be explained by detection bias, as people are likely to start a statin during a period of time when their health is of concern; they will tend to see their general practitioner more often and may be more likely to have a blood glucose test, thereby increasing the likelihood of detection of diabetes. Ascertainment bias might also partly account for the increased risk observed, particularly in the first year of starting treatment, because people prescribed statins may consult their general practitioner more often and have more blood tests, thereby increasing the likelihood of detection of abnormal blood glucose levels.

We also recognized the potential for survival bias in our study, as the mean follow up time was different between groups (5.43 years for statin users and 3.89 years for nonusers) and statin users were slightly less likely to die (10.79%) compared to the nonusers group (11.66%). This means that statin nonusers might die earlier and, consequently, don’t have the same probability of developing T2DM as the statin users who remain under follow up. This could bias the estimate of effect for statin use away from the null.

### Implications for clinical practice and future research

Our observational study provides evidence that statin use is associated with an increased risk of T2DM development, which increases with longer duration of use and higher baseline BMI levels. The increased risk was greater than that observed in randomized trials of statin therapy. The increased risk was less marked among people with diagnosed hypertension or cardiovascular disease. The Cholesterol Treatment Trialists’ collaboration has provided strong evidence of reductions in major vascular events from statins across all risk categories, with rates in diabetic populations comparable with those without diabetes [3-6]. However, in a recent study using the Taiwan National Health Insurance Research Database, incident diabetes after statin therapy was associated with an increased risk of major cardiovascular events and in-hospital death compared with the nondiabetic controls, although not in the high-risk and secondary prevention cohorts [15]. Based on these findings, the authors suggest caution in extending statin therapy to low-risk individuals, as has been advocated recently [15].

The consequences of statin-induced T2DM deserve more attention. Particularly, since findings from a recent cohort of 3.8 million general population patients showed substantive overuse of statins in low CVD risk and underuse in high CVD risk [50]. Once a patient develops diabetes, although their cardiovascular risk doubles, statins prevent cardiovascular events. Among a low risk population (5-year cardiovascular risk less than 10%), the Cholesterol Treatment Trialists’ collaboration estimated that 11 major vascular events would be avoided per 1000 individuals treated for 5 years [5]. For longer periods of treatment both the increased risk of T2DM and the protective effect against vascular events increased. Further research should explore the mechanism underlying statin induced T2DM.

## Conclusion

Statin use is associated with an increased risk of T2DM, which increases with longer duration of use and higher baseline BMI levels. Our results suggest that the relative risk is higher among people without diagnosed hypertension or cardiovascular disease.

These findings should be interpreted with caution as observational studies are subject to residual confounding by indication and other biases that cannot be ruled out.

## Abbreviations

BMI: Body Mass Index; CVD: Cardiovascular Diseases; CPRD: Clinical Practice Research Datalink; MHRA: Medicines & Health-products Regulatory Agency; PS: Propensity Scores; RCT: Randomised Controlled Trial; T2DM: Type 2 Diabetes Mellitus; UK: United Kingdom.

## Competing interests

AFM, HF and SE have no relevant interests to declare. LS has undertaken advisory work for GSK. ID holds stock in and consults for GSK, and consults for Gilead.

## Authors’ contributions

SE origination of idea and control of content. All authors were involved in the design of the study and contributed to interpretation of results and drafting of the first submission. AFM carried out the main data management and statistical analysis, with the help of ID and HF. All authors read and approved the final manuscript.

## Pre-publication history

The pre-publication history for this paper can be accessed here:

http://www.biomedcentral.com/1471-2261/14/85/prepub

## Supplementary Material

Additional file 1 **List S1- Read codes for type 2 diabetes. Table S1.** Sensitivity analysis censoring observation periods at time of stopping or changing therapy. **Table S2.** Baseline characteristics (%) of hypertensive and CVD patients, according to exposure status. **Table S3.** Incident rates of T2DM at different follow-up times, according to hypertension and CVD diagnosis, for individuals with BMI ≤ 25Kg/m^2^. Click here for file
