# Supplementary material for: Statins and the risk of type 2 diabetes mellitus: cohort study using the UK clinical practice pesearch datalink
Source: BMC Cardiovasc Disord. 2014 Jul 15;14:85. doi: 10.1186/1471-2261-14-85 (PMC4118294; doi:10.1186/1471-2261-14-85)
Supplement: Additional file 1 — List S1- Read codes for type 2 diabetes. Table S1. Sensitivity analysis censoring observation periods at time of stopping or changing therapy. Table S2. Baseline characteristics (%) of hypertensive and CVD patients, according to exposure status. Table S3. Incident rates of T2DM at different follow-up times, according to hypertension and CVD diagnosis, for individuals with BMI ≤ 25Kg/m2. [file 1471-2261-14-85-S1.doc]

**Additional files:**

**List S1**- Read codes for type 2 diabetes

**Table S1** - Sensitivity analysis censoring observation periods at time of stopping or changing therapy.

**Table S2** - Baseline characteristics (%) of hypertensive and CVD patients, according to exposure status

**Table S3** - Incident rates of T2DM at different follow-up times, according to hypertension and CVD diagnosis, for individuals with BMI≤25Kg/m^2^

**Read codes for type 2 diabetes -** list S1

| 9OL..00 | Diabetes monitoring admin. |
| --- | --- |
| 66A..00 | Diabetic monitoring |
| 66AS.00 | Diabetic annual review |
| C10..00 | Diabetes mellitus |
| 9N1Q.00 | Seen in diabetic clinic |
| C10F.00 | Type 2 diabetes mellitus |
| 9OL4.00 | Diabetes monitoring 1st letter |
| 66A2.00 | Follow-up diabetic assessment |
| 9NND.00 | Under care of diabetic foot screener |
| 66AP.00 | Diabetes: practice programme |
| 9OL1.00 | Attends diabetes monitoring |
| 66A4.00 | Diabetic on oral treatment |
| 68A7.00 | Diabetic retinopathy screening |
| 66AZ.00 | Diabetic monitoring NOS |
| 66AJ.00 | Diabetic - poor control |
| C100112 | Non-insulin dependent diabetes mellitus |
| 66Ac.00 | Diabetic peripheral neuropathy screening |
| 9OL5.00 | Diabetes monitoring 2nd letter |
| 66A3.00 | Diabetic on diet only |
| 66Aq.00 | Diabetic foot screen |
| C109.00 | Non-insulin dependent diabetes mellitus |
| F420.00 | Diabetic retinopathy |
| 13B1.00 | Diabetic diet |
| 9OLA.00 | Diabetes monitor. check done |
| 7L19800 | Subcutaneous injection of insulin |
| 66AR.00 | Diabetes management plan given |
| 9h42.00 | Excepted from diabetes quality indicators: Informed dissent |
| 2G5E.00 | O/E - Right diabetic foot at low risk |
| 66A5.00 | Diabetic on insulin |
| 2G5I.00 | O/E - Left diabetic foot at low risk |
| 9h41.00 | Excepted from diabetes qual indicators: Patient unsuitable |
| F420000 | Background diabetic retinopathy |
| 9N4I.00 | DNA - Did not attend diabetic clinic |
| 66AI.00 | Diabetic - good control |
| C109.12 | Type 2 diabetes mellitus |
| 66AQ.00 | Diabetes: shared care programme |
| 66AD.00 | Fundoscopy - diabetic check |
| 8BL2.00 | Patient on maximal tolerated therapy for diabetes |
| 1434.00 | H/O: diabetes mellitus |
| 9OL6.00 | Diabetes monitoring 3rd letter |
| 66Ai.00 | Diabetic 6 month review |
| 9N1v.00 | Seen in diabetic eye clinic |
| 8CA4100 | Pt advised re diabetic diet |
| 2BBP.00 | O/E - right eye background diabetic retinopathy |
| 2BBQ.00 | O/E - left eye background diabetic retinopathy |
| 8B3l.00 | Diabetes medication review |
| 66A9.00 | Understands diet - diabetes |
| 66Ab.00 | Diabetic foot examination |
| 42W..00 | Hb. A1C - diabetic control |
| 66AU.00 | Diabetes care by hospital only |
| 9NM0.00 | Attending diabetes clinic |
| 8H7r.00 | Refer to diabetic foot screener |
| 8I3X.00 | Diabetic retinopathy screening refused |
| 2G5F.00 | O/E - Right diabetic foot at moderate risk |
| 2G5J.00 | O/E - Left diabetic foot at moderate risk |
| 66A1.00 | Initial diabetic assessment |
| F420400 | Diabetic maculopathy |
| 66AW.00 | Diabetic foot risk assessment |
| 9OLA.11 | Diabetes monitored |
| 13AB.00 | Diabetic lipid lowering diet |
| 2BBM.00 | O/E - diabetic maculopathy absent both eyes |
| ZC2C800 | Dietary advice for diabetes mellitus |
| C100100 | Diabetes mellitus, adult onset, no mention of complication |
| 66Ao.00 | Diabetes type 2 review |
| 9OL8.00 | Diabetes monitor.phone invite |
| 9N4p.00 | Did not attend diabetic retinopathy clinic |
| 9N1i.00 | Seen in diabetic foot clinic |
| 14P3.00 | H/O: insulin therapy |
| C101.00 | Diabetes mellitus with ketoacidosis |
| 66A8.00 | Has seen dietician - diabetes |
| 66AT.00 | Annual diabetic blood test |
| C10FJ00 | Insulin treated Type 2 diabetes mellitus |
| 9OL3.00 | Diabetes monitoring default |
| 66AV.00 | Diabetic on insulin and oral treatment |
| 66A7.00 | Frequency of hypo. attacks |
| 9OL..11 | Diabetes clinic administration |
| 66AA.11 | Injection sites - diabetic |
| 66AH000 | Conversion to insulin |
| 8CS0.00 | Diabetes care plan agreed |
| 2G5G.00 | O/E - Right diabetic foot at high risk |
| 66AH.00 | Diabetic treatment changed |
| 2G5K.00 | O/E - Left diabetic foot at high risk |
| 9OLD.00 | Diabetic patient unsuitable for digital retinal photography |
| F372.12 | Diabetic neuropathy |
| F420600 | Non proliferative diabetic retinopathy |
| C106.12 | Diabetes mellitus with neuropathy |
| 9OL7.00 | Diabetes monitor.verbal invite |
| C106.00 | Diabetes mellitus with neurological manifestation |
| 8H4F.00 | Referral to diabetologist |
| 8I3W.00 | Diabetic foot examination declined |
| 68A9.00 | Diabetic retinopathy screening offered |
| 13AC.00 | Diabetic weight reducing diet |
| F420100 | Proliferative diabetic retinopathy |
| 66AY.00 | Diabetic diet - good compliance |
| C10FM00 | Type 2 diabetes mellitus with persistent microalbuminuria |
| C109.13 | Type II diabetes mellitus |
| C109.11 | NIDDM - Non-insulin dependent diabetes mellitus |
| 9OLZ.00 | Diabetes monitoring admin.NOS |
| 2G5A.00 | O/E - Right diabetic foot at risk |
| 2BBW.00 | O/E - right eye diabetic maculopathy |
| 2BBX.00 | O/E - left eye diabetic maculopathy |
| 2G5B.00 | O/E - Left diabetic foot at risk |
| 8HBG.00 | Diabetic retinopathy 12 month review |
| 8H7C.00 | Refer, diabetic liaison nurse |
| C109J00 | Insulin treated Type 2 diabetes mellitus |
| 66Af.00 | Patient diabetes education review |
| 66A6.00 | Last hypo. attack |
| ZLA2500 | Seen by diabetic liaison nurse |
| 8Hj0.00 | Referral to diabetes structured education programme |
| 7276.00 | Pan retinal photocoagulation for diabetes |
| C10FL00 | Type 2 diabetes mellitus with persistent proteinuria |
| 8Hl1.00 | Referral for diabetic retinopathy screening |
| 68AB.00 | Diabetic digital retinopathy screening offered |
| 8Hj4.00 | Referral to DESMOND diabetes structured education programme |
| F420200 | Preproliferative diabetic retinopathy |
| C104.11 | Diabetic nephropathy |
| 8HTk.00 | Referral to diabetic eye clinic |
| 9h4..00 | Exception reporting: diabetes quality indicators |
| C100.00 | Diabetes mellitus with no mention of complication |
| 2BBL.00 | O/E - diabetic maculopathy present both eyes |
| 66AM.00 | Diabetic - follow-up default |
| 9N2i.00 | Seen by diabetic liaison nurse |
| C10F.11 | Type II diabetes mellitus |
| M271200 | Mixed diabetic ulcer - foot |
| 2BBR.00 | O/E - right eye preproliferative diabetic retinopathy |
| 66Ae.00 | HbA1c target |
| 2BBS.00 | O/E - left eye preproliferative diabetic retinopathy |
| 8H7f.00 | Referral to diabetes nurse |
| 42c..00 | HbA1 - diabetic control |
| M271000 | Ischaemic ulcer diabetic foot |
| 679R.00 | Patient offered diabetes structured education programme |
| F171100 | Autonomic neuropathy due to diabetes |
| 66Am.00 | Insulin dose changed |
| ZL62500 | Referral to diabetes nurse |
| C109700 | Non-insulin dependent diabetes mellitus - poor control |
| F464000 | Diabetic cataract |
| F420z00 | Diabetic retinopathy NOS |
| C10FC00 | Type 2 diabetes mellitus with nephropathy |
| C105.00 | Diabetes mellitus with ophthalmic manifestation |
| 8Hj5.00 | Referral to XPERT diabetes structured education programme |
| 2BBT.00 | O/E - right eye proliferative diabetic retinopathy |
| C10F600 | Type 2 diabetes mellitus with retinopathy |
| M271100 | Neuropathic diabetic ulcer - foot |
| 8Hl4.00 | Referral to community diabetes specialist nurse |
| 2BBV.00 | O/E - left eye proliferative diabetic retinopathy |
| C104.00 | Diabetes mellitus with renal manifestation |
| 9N0m.00 | Seen in diabetic nurse consultant clinic |
| 9OLB.00 | Attended diabetes structured education programme |
| 9NN9.00 | Under care of diabetes specialist nurse |
| 66Aa.00 | Diabetic diet - poor compliance |
| C107.00 | Diabetes mellitus with peripheral circulatory disorder |
| F372.00 | Polyneuropathy in diabetes |
| 66AJz00 | Diabetic - poor control NOS |
| 9N2d.00 | Seen by diabetologist |
| 8H2J.00 | Admit diabetic emergency |
| 2BBF.00 | Retinal abnormality - diabetes related |
| 9OL2.00 | Refuses diabetes monitoring |
| 66Ad.00 | Hypoglycaemic attack requiring 3rd party assistance |
| 8A13.00 | Diabetic stabilisation |
| C10F700 | Type 2 diabetes mellitus - poor control |
| 9N0n.00 | Seen in community diabetes specialist clinic |
| 93C4.00 | Patient consent given for addition to diabetic register |
| 66AJ.11 | Unstable diabetes |
| 9M00.00 | Informed consent for diabetes national audit |
| F420300 | Advanced diabetic maculopathy |
| C10F900 | Type 2 diabetes mellitus without complication |
| 66AJ000 | Chronic hyperglycaemia |
| 66AK.00 | Diabetic - cooperative patient |
| 2G5C.00 | Foot abnormality - diabetes related |
| 66Ap.00 | Insulin treatment initiated |
| 9N0o.00 | Seen in community diabetic specialist nurse clinic |
| 9NN8.00 | Under care of diabetologist |
| ZL62600 | Referral to diabetic liaison nurse |
| C100z00 | Diabetes mellitus NOS with no mention of complication |
| N030100 | Diabetic Charcot arthropathy |
| F381311 | Diabetic amyotrophy |
| TJ23A00 | Adverse reaction to metformin hydrochloride |
| M037200 | Cellulitis in diabetic foot |
| C10FN00 | Type 2 diabetes mellitus with ketoacidosis |
| 66AN.00 | Date diabetic treatment start |
| 8HHy.00 | Referral to diabetic register |
| 2G5H.00 | O/E - Right diabetic foot - ulcerated |
| 2G51000 | Foot abnormality - diabetes related |
| 2G5L.00 | O/E - Left diabetic foot - ulcerated |
| 66Ak.00 | Diabetic monitoring - lower risk albumin excretion |
| 9OLM.00 | Diabetes structured education programme declined |
| 8CR2.00 | Diabetes clinical management plan |
| C10FK00 | Hyperosmolar non-ketotic state in type 2 diabetes mellitus |
| 66A7100 | Frequency of GP or paramedic treated hypoglycaemia |
| ZV65312 | [V]Dietary counselling in diabetes mellitus |
| C103.00 | Diabetes mellitus with ketoacidotic coma |
| ZRB6.00 | Diabetes wellbeing questionnaire |
| 66AJ200 | Loss of hypoglycaemic warning |
| 9OLK.00 | DESMOND diabetes structured education programme completed |
| C104z00 | Diabetes mellitus with nephropathy NOS |
| F372100 | Chronic painful diabetic neuropathy |
| F3y0.00 | Diabetic mononeuropathy |
| C107.11 | Diabetes mellitus with gangrene |
| F372.11 | Diabetic polyneuropathy |
| 66AL.00 | Diabetic-uncooperative patient |
| 9kL..00 | Insulin initiation - enhanced services administration |
| 9OLL.00 | XPERT diabetes structured education programme completed |
| C109400 | Non-insulin dependent diabetes mellitus with ulcer |
| C10D.00 | Diabetes mellitus autosomal dominant type 2 |
| M21yC00 | Insulin lipohypertrophy |
| L180500 | Pre-existing diabetes mellitus, insulin-dependent |
| C109900 | Non-insulin-dependent diabetes mellitus without complication |
| 66A7000 | Frequency of hospital treated hypoglycaemia |
| 8H4e.00 | Referral to diabetes special interest general practitioner |
| C106z00 | Diabetes mellitus NOS with neurological manifestation |
| C102.00 | Diabetes mellitus with hyperosmolar coma |
| K01x100 | Nephrotic syndrome in diabetes mellitus |
| 66AJ300 | Recurrent severe hypos |
| C101z00 | Diabetes mellitus NOS with ketoacidosis |
| G73y000 | Diabetic peripheral angiopathy |
| 66Al.00 | Diabetic monitoring - higher risk albumin excretion |
| ZRbH.00 | Perceived control of insulin-dependent diabetes |
| N030000 | Diabetic cheiroarthropathy |
| 8A12.00 | Diabetic crisis monitoring |
| C105z00 | Diabetes mellitus NOS with ophthalmic manifestation |
| 9OLN.00 | Diabetes monitor invitation by SMS (short message service) |
| 8HBH.00 | Diabetic retinopathy 6 month review |
| ZL22500 | Under care of diabetic liaison nurse |
| 9OLF.00 | Diabetes structured education programme completed |
| F35z000 | Diabetic mononeuritis NOS |
| 8H3O.00 | Non-urgent diabetic admission |
| C10FQ00 | Type 2 diabetes mellitus with exudative maculopathy |
| C10G.00 | Secondary pancreatic diabetes mellitus |
| C10F000 | Type 2 diabetes mellitus with renal complications |
| C10F911 | Type II diabetes mellitus without complication |
| C106100 | Diabetes mellitus, adult onset, + neurological manifestation |
| C10FB00 | Type 2 diabetes mellitus with polyneuropathy |
| C10F200 | Type 2 diabetes mellitus with neurological complications |
| 7L10000 | Continuous subcutaneous infusion of insulin |
| 66AG.00 | Diabetic drug side effects |
| 8HLE.00 | Diabetology D.V. done |
| C10FH00 | Type 2 diabetes mellitus with neuropathic arthropathy |
| F372200 | Asymptomatic diabetic neuropathy |
| 9OLG.00 | Attended XPERT diabetes structured education programme |
| C109600 | Non-insulin-dependent diabetes mellitus with retinopathy |
| U60231C | [X] Adverse reaction to metformin hydrochloride |
| 8Hg4.00 | Discharged from care of diabetes specialist nurse |
| F420500 | Advanced diabetic retinal disease |
| 9OL9.00 | Diabetes monitoring deleted |
| M21yC11 | Insulin site lipohypertrophy |
| C10FE00 | Type 2 diabetes mellitus with diabetic cataract |
| 2BBk.00 | O/E - right eye stable treated prolif diabetic retinopathy |
| F372000 | Acute painful diabetic neuropathy |
| 66AO.00 | Date diabetic treatment stopp. |
| TJ23400 | Adverse reaction to gliclazide |
| 8I3k.00 | Insulin therapy declined |
| C105100 | Diabetes mellitus, adult onset, + ophthalmic manifestation |
| Cyu2.00 | [X]Diabetes mellitus |
| 9OLJ.00 | DAFNE diabetes structured education programme completed |
| C107.12 | Diabetes with gangrene |
| C10zz00 | Diabetes mellitus NOS with unspecified complication |
| 44V3.00 | Glucose tol. test diabetic |
| C10F400 | Type 2 diabetes mellitus with ulcer |
| C10FJ11 | Insulin treated Type II diabetes mellitus |
| 42WZ.00 | Hb. A1C - diabetic control NOS |
| ZLD7500 | Discharge by diabetic liaison nurse |
| C107z00 | Diabetes mellitus NOS with peripheral circulatory disorder |
| C10K.00 | Type A insulin resistance |
| C10A100 | Malnutrition-related diabetes mellitus with ketoacidosis |
| ZV6DA00 | [V]Admitted for commencement of insulin |
| C101000 | Diabetes mellitus, juvenile type, with ketoacidosis |
| 2BBl.00 | O/E - left eye stable treated prolif diabetic retinopathy |
| 9OLH.00 | Attended DAFNE diabetes structured education programme |
| C106.13 | Diabetes mellitus with polyneuropathy |
| R054200 | [D]Gangrene of toe in diabetic |
| C10y.00 | Diabetes mellitus with other specified manifestation |
| C10F711 | Type II diabetes mellitus - poor control |
| C10FL11 | Type II diabetes mellitus with persistent proteinuria |
| U602300 | [X]Insul/oral hypoglyc drugs caus adverse eff therapeut use |
| C10z.00 | Diabetes mellitus with unspecified complication |
| C109C00 | Non-insulin dependent diabetes mellitus with nephropathy |
| C10F100 | Type 2 diabetes mellitus with ophthalmic complications |
| 679L000 | Education in self management of diabetes |
| C10z100 | Diabetes mellitus, adult onset, + unspecified complication |
| C10FR00 | Type 2 diabetes mellitus with gastroparesis |
| 8Hj3.00 | Referral to DAFNE diabetes structured education programme |
| C10FA00 | Type 2 diabetes mellitus with mononeuropathy |
| C10FD00 | Type 2 diabetes mellitus with hypoglycaemic coma |
| 2G5W.00 | O/E - left chronic diabetic foot ulcer |
| F440700 | Diabetic iritis |
| ZC2CA00 | Dietary advice for type II diabetes |
| C10A.00 | Malnutrition-related diabetes mellitus |
| C10N.00 | Secondary diabetes mellitus |
| R054300 | [D]Widespread diabetic foot gangrene |
| C104100 | Diabetes mellitus, adult onset, with renal manifestation |
| C10N100 | Cystic fibrosis related diabetes mellitus |
| F345000 | Diabetic mononeuritis multiplex |
| C109711 | Type II diabetes mellitus - poor control |
| C107200 | Diabetes mellitus, adult with gangrene |
| C10F500 | Type 2 diabetes mellitus with gangrene |
| 2BBo.00 | O/E - sight threatening diabetic retinopathy |
| C101100 | Diabetes mellitus, adult onset, with ketoacidosis |
| C109712 | Type 2 diabetes mellitus - poor control |
| C107100 | Diabetes mellitus, adult, + peripheral circulatory disorder |
| 8HVU.00 | Private referral to diabetologist |
| C109J12 | Insulin treated Type II diabetes mellitus |
| F381300 | Myasthenic syndrome due to diabetic amyotrophy |
| ZV6DB00 | [V]Admitted for conversion to insulin |
| 8HTi.00 | Referral to multidisciplinary diabetic clinic |
| 66Ae000 | HbA1c target level - IFCC standardised |
| TJ23000 | Adverse reaction to insulins |
| F420700 | High risk proliferative diabetic retinopathy |
| C10F611 | Type II diabetes mellitus with retinopathy |
| 2G5V.00 | O/E - right chronic diabetic foot ulcer |
| C109K00 | Hyperosmolar non-ketotic state in type 2 diabetes mellitus |
| C102100 | Diabetes mellitus, adult onset, with hyperosmolar coma |
| C10FF00 | Type 2 diabetes mellitus with peripheral angiopathy |
| F420800 | High risk non proliferative diabetic retinopathy |
| C107400 | NIDDM with peripheral circulatory disorder |
| C10F300 | Type 2 diabetes mellitus with multiple complications |
| 8CP2.00 | Transition of diabetes care options discussed |
| N030011 | Diabetic cheiropathy |
| 66At.00 | Diabetic dietary review |
| C10M.00 | Lipoatrophic diabetes mellitus |
| 9M10.00 | Informed dissent for diabetes national audit |
| C10FP00 | Type 2 diabetes mellitus with ketoacidotic coma |
| C109611 | Type II diabetes mellitus with retinopathy |
| C10FG00 | Type 2 diabetes mellitus with arthropathy |
| C109J11 | Insulin treated non-insulin dependent diabetes mellitus |
| C101y00 | Other specified diabetes mellitus with ketoacidosis |
| C109411 | Type II diabetes mellitus with ulcer |
| 8I2S.00 | Glitazones contraindicated |
| C109000 | Non-insulin-dependent diabetes mellitus with renal comps |
| C109100 | Non-insulin-dependent diabetes mellitus with ophthalm comps |
| C106y00 | Other specified diabetes mellitus with neurological comps |
| TJ23.00 | Adverse reaction to insulins and antidiabetic agents |
| 9h43.00 | Excepted from diabetes qual indicators: service unavailable |
| C106.11 | Diabetic amyotrophy |
| TJ23z00 | Adverse reaction to insulins and antidiabetic agents NOS |
| C104y00 | Other specified diabetes mellitus with renal complications |
| C109612 | Type 2 diabetes mellitus with retinopathy |
| C109212 | Type 2 diabetes mellitus with neurological complications |
| C109200 | Non-insulin-dependent diabetes mellitus with neuro comps |
| C106000 | Diabetes mellitus, juvenile, + neurological manifestation |
| ZRB4.00 | Diabetes clinic satisfaction questionnaire |
| C109412 | Type 2 diabetes mellitus with ulcer |
| ZRB5.11 | DTSQ - Diabetes treatment satisfaction questionnaire |
| C109E00 | Non-insulin depend diabetes mellitus with diabetic cataract |
| 8I2P.00 | Sulphonylureas contraindicated |
| C109B00 | Non-insulin dependent diabetes mellitus with polyneuropathy |
| C109E11 | Type II diabetes mellitus with diabetic cataract |
| 8HKE.00 | Diabetology D.V. requested |
| C10yz00 | Diabetes mellitus NOS with other specified manifestation |
| C109C12 | Type 2 diabetes mellitus with nephropathy |
| C109500 | Non-insulin dependent diabetes mellitus with gangrene |
| C109H00 | Non-insulin dependent d m with neuropathic arthropathy |
| C109D00 | Non-insulin dependent diabetes mellitus with hypoglyca coma |
| C109011 | Type II diabetes mellitus with renal complications |
| C109300 | Non-insulin-dependent diabetes mellitus with multiple comps |
| U602312 | [X] Adverse reaction to insulins |
| C10zy00 | Other specified diabetes mellitus with unspecified comps |
| U602311 | [X] Adverse reaction to insulins and antidiabetic agents |
| TJ23500 | Adverse reaction to glipizide |
| 66Ar.00 | Insulin treatment stopped |
| C109H11 | Type II diabetes mellitus with neuropathic arthropathy |
| 889A.00 | Diab mellit insulin-glucose infus acute myocardial infarct |
| C109H12 | Type 2 diabetes mellitus with neuropathic arthropathy |
| 9NiC.00 | Did not attend DAFNE diabetes structured education programme |
| 66AQ100 | Declined consent for diabetes year of care programme |
| C10yy00 | Other specified diabetes mellitus with other spec comps |
| C10F311 | Type II diabetes mellitus with multiple complications |
| C109E12 | Type 2 diabetes mellitus with diabetic cataract |
| C108y00 | Other specified diabetes mellitus with multiple comps |
| TJ23300 | Adverse reaction to glibenclamide |
| C109511 | Type II diabetes mellitus with gangrene |
| C10y100 | Diabetes mellitus, adult, + other specified manifestation |
| C10N000 | Secondary diabetes mellitus without complication |
| 66As.00 | Diabetic on subcutaneous treatment |
| 66At100 | Type II diabetic dietary review |
| C109012 | Type 2 diabetes mellitus with renal complications |
| C109D12 | Type 2 diabetes mellitus with hypoglycaemic coma |
| U602316 | [X] Adverse reaction to gliclazide |
| C102z00 | Diabetes mellitus NOS with hyperosmolar coma |
| C109512 | Type 2 diabetes mellitus with gangrene |
| C109F00 | Non-insulin-dependent d m with peripheral angiopath |
| C109C11 | Type II diabetes mellitus with nephropathy |
| C103z00 | Diabetes mellitus NOS with ketoacidotic coma |
| 9N1o.00 | Seen in multidisciplinary diabetic clinic |
| C109G00 | Non-insulin dependent diabetes mellitus with arthropathy |
| C105y00 | Other specified diabetes mellitus with ophthalmic complicatn |
| C109F11 | Type II diabetes mellitus with peripheral angiopathy |
| C10FM11 | Type II diabetes mellitus with persistent microalbuminuria |
| C10F411 | Type II diabetes mellitus with ulcer |
| 66Au.00 | Diabetic erectile dysfunction review |
| 66Av.00 | Diabetic assessment of erectile dysfunction |
| Cyu2000 | [X]Other specified diabetes mellitus |
| C109B11 | Type II diabetes mellitus with polyneuropathy |
| C10F011 | Type II diabetes mellitus with renal complications |
| C103y00 | Other specified diabetes mellitus with coma |
| C109211 | Type II diabetes mellitus with neurological complications |
| ZRB4.11 | CSQ - Diabetes clinic satisfaction questionnaire |
| 3883.00 | Diabetes treatment satisfaction questionnaire |
| 8IAs.00 | Diabetic dietary review declined |
| 2BBr.00 | Impaired vision due to diabetic retinopathy |
| TJ23900 | Adverse reaction to tolbutamide |
| C10FB11 | Type II diabetes mellitus with polyneuropathy |
| C109D11 | Type II diabetes mellitus with hypoglycaemic coma |
| C109111 | Type II diabetes mellitus with ophthalmic complications |
| C109F12 | Type 2 diabetes mellitus with peripheral angiopathy |
| C10A000 | Malnutrition-related diabetes mellitus with coma |
| C103100 | Diabetes mellitus, adult onset, with ketoacidotic coma |
| C109A00 | Non-insulin dependent diabetes mellitus with mononeuropathy |
| 8HME.00 | Listed for Diabetology admissn |
| C10FA11 | Type II diabetes mellitus with mononeuropathy |
| U60231B | [X] Adverse reaction to tolbutamide |
| U60231E | [X] Adverse reaction to insulins and antidiabetic agents NOS |
| Cyu2300 | [X]Unspecified diabetes mellitus with renal complications |
| 8HgC.00 | Discharged from diabetes shared care programme |
| 66AQ000 | Unsuitable for diabetes year of care programme |
| C10F111 | Type II diabetes mellitus with ophthalmic complications |
| TJ23B00 | Adverse reaction to glucagon |
| C109G11 | Type II diabetes mellitus with arthropathy |
| U602315 | [X] Adverse reaction to glibenclamide |
| C109G12 | Type 2 diabetes mellitus with arthropathy |
| C109A11 | Type II diabetes mellitus with mononeuropathy |
| C10K000 | Type A insulin resistance without complication |
| U60231A | [X] Adverse reaction to tolazamide |
| C108z00 | Unspecified diabetes mellitus with multiple complications |
| C109112 | Type 2 diabetes mellitus with ophthalmic complications |
| U602318 | [X] Adverse reaction to gliquidone |
| TJ23200 | Adverse reaction to chlorpropamide |
| C10FE11 | Type II diabetes mellitus with diabetic cataract |
| U602317 | [X] Adverse reaction to glipzide |
| C10G000 | Secondary pancreatic diabetes mellitus without complication |
| ZRB6.11 | DWBQ - Diabetes wellbeing questionnaire |
| C10F211 | Type II diabetes mellitus with neurological complications |
| C10E512 | Insulin dependent diabetes mellitus with ulcer |
| C10FD11 | Type II diabetes mellitus with hypoglycaemic coma |
| SL23100 | Biguanide poisoning |
| Kyu0300 | [X]Glomerular disorders in diabetes mellitus |
| C10A500 | Malnutritn-relat diabetes melitus wth periph circul complctn |
| C10FC11 | Type II diabetes mellitus with nephropathy |
| 66Aw.00 | Insulin dose |
| TJ23800 | Adverse reaction to tolazamide |

**Table S1 - Sensitivity analysis censoring observation periods at time of stopping or changing therapy**

| **Follow up time (years) Ps [5-95%] (N=1,448,993)** | **Hazard ratio (95% CI)*** | | **Incident T2DM per 1000 person-years** | | | |
| --- | --- | --- | --- | --- | --- | --- |
|  | **STOP** | **CHANGE** | **STOP** | | **CHANGE** | |
|  |  |  | **Exposed** | **Unexposed** | **Exposed** | **Unexposed** |
| **No Hypertension** | | | | | | |
| **0-1 yr** | 2.35 (2.25-2.46) | 2.97 (2.86-3.09) | 22.07 (21.46-22.70) | 8.65 (8.44-8.85) | 29.71 (28.99-30.44) | 8.91 (8.70-9.11) |
| **1-3 yr** | 1.62 (1.57-1.68) | 1.89 (1.83-1.95) | 20.07 (19.63-20.53) | 10.60 (10.42-10.77) | 23.83 (23.33-24.33) | 10.68 (10.49-10.86) |
| **3-5 yr** | 2.14 (2.06-2.24) | 2.33 (2.23-2.43) | 23.55 (22.98-24.14) | 9.56 (9.33-9.80) | 26.31 (25.66-26.98) | 9.64 (9.41-9.88) |
| **5-10 yr** | 2.66 (2.55-2.79) | 2.78 (2.65-2.92) | 29.32 (28.70-29.96) | 9.32 (9.06-9.59) | 30.84 (30.10-31.59) | 9.37 (9.11-9.65) |
| **10-15 yr** | 3.31 (2.90-3.78) | 3.09 (2.66-3.59) | 34.17 (32.48-35.96) | 9.31 (8.48-10.21) | 34.59 (32.37-36.96) | 9.34 (8.50-10.26) |
| **15-20 yr** | 4.37 (2.86-6.67) | 4.20 (2.57-6.86) | 44.52 (38.06-52.09) | 11.55 (8.41-15.88) | 45.38 (36.14-56.99) | 11.72 (8.49-16.17) |
| **Hypertension** | | | | | | |
| **0-1 yr** | 1.08 (1.02-1.14) | 1.31 (1.25-1.38) | 23.11 (19.86-20.96) | 20.40 (19.86-20.96) | 31.15 (30.26-32.07) | 21.28 (20.72-21.85) |
| **1-3 yr** | 0.90 (0.86-0.94) | 1.01 (0.97-1.05) | 23.67 (23.09-24.72) | 24.20 (23.68-24.72) | 27.86 (27.21-28.54) | 24.76 (24.23-25.30) |
| **3-5 yr** | 1.29 (1.22-1.37) | 1.39 (1.31-1.47) | 29.80 (29.01-30.61) | 20.51 (19.82-21.23) | 32.72 (31.83-33.63) | 20.96 (20.24-21.70) |
| **5-10 yr** | 1.64 (1.53-1.75) | 1.62 (1.51-1.75) | 35.80 (34.90-36.73) | 19.25 (18.38-20.17) | 37.02 (35.97-38.10) | 19.70 (18.79-20.65) |
| **10-15 yr** | 2.26 (1.73-2.95) | 2.19 (1.64-2.93) | 41.68 (38.76-44.83) | 18.33 (15.02-22.37) | 42.79 (38.98-46.97) | 19.15 (15.64-23.44) |
| **15-20 yr** | 2.66 (0.89-7.97) | 1.09 (0.22-5.51) | 47.97 (36.25-63.47) | 22.38 (9.31-53.77) | 26.73 (16.12-44.35) | 19.21 (7.21-51.17) |
| **No CVD** | | | | | | |
| **0-1 yr** | 1.99 (1.92-2.06) | 2.49 (2.41-2.58) | 23.47 (22.92-24.03) | 10.74 (10.54-10.95) | 31.35 (30.71-32.00) | 11.04 (10.83-11.25) |
| **1-3 yr** | 1.42 (1.38-1.46) | 1.63 (1.58-1.67) | 21.90 (21.50-22.32) | 12.93 (12.75-13.11) | 25.44 (24.99-25.90) | 13.05 (12.87-13.24) |
| **3-5 yr** | 1.98 (1.91-2.05) | 2.12 (2.04-2.20) | 26.58 (26.04-27.14) | 11.18 (10.95-11.42) | 28.83 (28.23-29.45) | 11.29 (11.06-11.53) |
| **5-10 yr** | 2.49 (2.39-2.59) | 2.53 (2.42-2.64) | 32.31 (31.69-32.94) | 10.56 (10.30-10.83) | 32.99 (32.27-33.72) | 10.62 (10.35-10.89) |
| **10-15 yr** | 3.14 (2.77-3.55) | 2.85 (2.47-3.29) | 35.31 (33.47-37.27) | 10.08 (9.26-10.99) | 33.97 (31.64-36.47) | 10.21 (9.37-11.13) |
| **15-20 yr** | 4.28 (2.83-6.46) | 3.80 (2.31-6.25) | 46.07 (39.13-54.25) | 11.81 (8.70-16.04) | 41.48 (32.47-52.99) | 11.71 (8.55-16.03) |
| **CVD** | | | | | | |
| **0-1 yr** | 0.83 (0.77-0.91) | 1.01 (0.94-1.09) | 18.85 (17.91-19.83) | 21.05 (20.08-22.07) | 26.31 (25.19-27.48) | 22.84 (21.81-23.91) |
| **1-3 yr** | 0.79 (0.74-0.85) | 0.95 (0.88-1.01) | 20.11 (19.38-20.88) | 23.77 (22.79-24.79) | 25.44 (24.58-26.32) | 24.54 (23.52-25.60) |
| **3-5 yr** | 1.02 (0.92-1.12) | 1.18 (1.07-1.30) | 24.24 (23.31-25.21) | 21.30 (19.83-22.88) | 28.96 (27.86-30.10) | 21.79 (20.25-23.45) |
| **5-10 yr** | 1.40 (1.25-1.57) | 1.51 (1.34-1.71) | 30.22 (29.29-31.17) | 18.73 (16.98-20.66) | 33.45 (32.31-34.63) | 19.71 (17.82-21.81) |
| **10-15 yr** | 2.26 (1.42-3.58) | 2.96 (1.73-5.05) | 37.96 (35.54-40.55) | 14.70 (9.49-22.79) | 42.06 (38.70-45.71) | 13.05 (7.87-21.65) |
| **15-20 yr** | 1.62 (0.35-7.51) | 1.29 (0.24-6.88) | 43.57 (33.90-56.01) | 48.47 (12.12-193.81) | 38.54 (26.04-57.03) | 57.40 (14.35-22.95) |

PS – propensity score; *Final model: adjusted for age and propensity score; CVD – cardiovascular disease; STOP – observation periods censored at time of statin withdrawn; CHANGE – observation periods censored at time exposure status changed.

**Table S**2 - Baseline characteristics (%) of hypertensive and CVD patients, according to exposure status

| **Baseline Characteristics**  **Pscore [5-95%]** | **Hypertension** | | **CVD** | |
| --- | --- | --- | --- | --- |
|  | **Exposed** | **Unexposed** | **Exposed** | **Unexposed** |
| **BMI>25Kg/m^2^** | 110,781 (71.73%) | 175,663 (67.04%) | 51,438 (63.46%) | 46,852 (56.94%) |
| **Hyperlipidaemia** | 9,782 (6.33%) | 11,771 (4.49%) | 4,134 (5.10%) | 4,108 (4.99%) |
| **Abnormal glucose** | 2,779 (1.80%) | 4,488 (1.71%) | 751 (0.93%) | 1,020 (1.24%) |
| **CVD** | 27,542 (17.83%) | 41,622 (15.88%) | - | - |
| **Hypertension** | - | - | 27,542 (33.98%) | 41,622 (50.58%) |

**Table S3 - Incident rates of T2DM at different follow-up times, according to hypertension and CVD diagnosis, for individuals with BMI≤25Kg/m^2^**

|  |  | **Statin use** | |
| --- | --- | --- | --- |
|  |  | **Yes** | **No** |
| **Follow up time (years) PS [5-95%]** | **Hazard ratio (95% CI)*** | **Incident T2DM per 1000 person-years (95% CI)** | **Incident T2DM per 1000 person-years (95% CI)** |
| **No Hypertension** | | | |
| **0-1 yr** | 1.81 (1.64-2.01) | 11.39 (10.62-12.22) | 5.20 (4.95-5.47) |
| **1-3 yr** | 1.39 (1.28-1.50) | 11.00 (10.43-11.60) | 6.05 (5.83-6.27) |
| **3-5 yr** | 1.62 (1.47-1.79) | 11.74 (11.04-12.47) | 5.42 (5.14-5.71) |
| **5-10 yr** | 2.12 (1.91-2.35) | 15.06 (14.30-15.87) | 5.15 (4.85-5.48) |
| **10-15 yr** | 2.59 (1.94-3.45) | 17.30 (15.35-19.50) | 5.38 (4.47-6.48) |
| **15-20 yr** | 2.10 (1.00-4.43) | 26.86 (19.78-36.48) | 10.92 (6.69-17.82) |
| **20-25 yr** | n.e. | 0 | 0 |
| **Hypertension** | | | |
| **0-1 yr** | 1.11 (0.97-1.28) | 12.58 (11.49-13.78) | 11.05 (10.31-11.85) |
| **1-3 yr** | 0.85 (0.76-0.95) | 11.99 (11.19-12.85) | 12.81 (12.11-13.54) |
| **3-5 yr** | 1.11 (0.95-1.29) | 14.86 (13.81-16.00) | 11.47 (10.52-12.49) |
| **5-10 yr** | 1.51 (1.27-1.81) | 17.24 (16.07-18.50) | 10.47 (9.33-11.75) |
| **10-15 yr** | 4.29 (2.11-8.70) | 21.19 (17.58-25.54) | 7.48 (4.24-13.16) |
| **15-20 yr** | n.e. | 21.13 (10.57-42.25) | 0 |
| **20-25 yr** | n.e. | 0 | 0 |
| **No CVD** | | | |
| **0-1 yr** | 1.75 (1.59-1.91) | 11.72 (10.99-12.50) | 5.79 (5.54-6.05) |
| **1-3 yr** | 1.27 (1.18-1.36) | 11.21 (10.67-11.76) | 6.74 (6.52-6.97) |
| **3-5 yr** | 1.55 (1.42-1.70) | 12.64 (11.97-13.35) | 6.04 (5.76-6.33) |
| **5-10 yr** | 2.05 (1.87-2.26) | 15.66 (14.89-16.47) | 5.63 (5.32-5.95) |
| **10-15 yr** | 2.77 (2.10-3.66) | 17.82 (15.67-20.27) | 5.44 (4.54-6.52) |
| **15-20 yr** | 2.53 (1.20-5.34) | 26.70 (19.26-37.02) | 9.75 (5.88-16.17) |
| **20-25 yr** | n.e. | 0 | 0 |
| **CVD** | | | |
| **0-1 yr** | 0.89 (0.75-1.07) | 12.09 (10.81-13.53) | 12.86 (11.60-14.26) |
| **1-3 yr** | 0.85 (0.73-0.98) | 11.80 (10.84-12.85) | 14.80 (13.53-16.18) |
| **3-5 yr** | 0.99 (0.80-1.22) | 13.39 (12.22-14.68) | 12.53 (10.75-14.61) |
| **5-10 yr** | 1.44 (1.11-1.86) | 16.01 (14.85-17.27) | 10.40 (8.39-12.90) |
| **10-15 yr** | 2.10 (0.83-5.35) | 19.04 (16.19-22.38) | 9.33 (3.89-22.41) |
| **15-20 yr** | 0.37 (0.33-4.04) | 23.35 (13.56-40.21) | 6.74 (9.49-47.85) |
| **20-25 yr** | n.e. | 0 | 0 |
